# No evidence for promoter region methylation of the succinate dehydrogenase and fumarate hydratase tumour suppressor genes in breast cancer

**DOI:** 10.1186/1756-0500-2-194

**Published:** 2009-09-25

**Authors:** Katie T Huang, Alexander Dobrovic, Stephen B Fox

**Affiliations:** 1 Molecular Pathology Research and Development Laboratory, Department of Pathology, Peter MacCallum Cancer Centre, Melbourne, Victoria 3002, Australia; 2 Departmant of Pathology, University of Melbourne, Parkville, Victoria 3010, Australia

## Abstract

**Background:**

Succinate dehydrogenase (SDH) and fumarate hydratase (FH) are tricarboxylic acid (TCA) cycle enzymes that are also known to act as tumour suppressor genes. Increased succinate or fumarate levels as a consequence of *SDH *and *FH *deficiency inhibit hypoxia inducible factor-1α (HIF-1α) prolyl hydroxylases leading to sustained HIF-1α expression in tumours. Since HIF-1α is frequently expressed in breast carcinomas, DNA methylation at the promoter regions of the *SDHA, SDHB, SDHC *and *SDHD *and *FH *genes was evaluated as a possible mechanism in silencing of *SDH *and *FH *expression in breast carcinomas.

**Findings:**

No DNA methylation was identified in the promoter regions of the *SDHA*, *SDHB*, *SDHC*, *SDHD *and *FH *genes in 72 breast carcinomas and 10 breast cancer cell lines using methylation-sensitive high resolution melting which detects both homogeneous and heterogeneous methylation.

**Conclusion:**

These results show that inactivation via DNA methylation of the promoter CpG islands of *SDH *and *FH *is unlikely to play a major role in sporadic breast carcinomas.

## Introduction

The hypoxia-inducible factor (HIF-1) transcription factor plays a pivotal role in breast tumour progression [1-4] by activating genes involved in angiogenesis, cell proliferation and survival [1,2,5]. Levels of HIF-1 α subunits (HIF-1α) are tightly regulated with rapid degradation via hydroxylation by prolyl hydroxylases (PHDs) 1, 2 and 3 and proteasomal degradation by the von Hippel-Lindau (VHL) protein [1]. Increased levels of fumarate and succinate inhibit PHD activity via product inhibition as well as by direct inhibition by competing with α-ketoglutarate at the PHD α-ketoglutarate binding site [6-8]. Thus any mechanism whereby the level of succinate dehydrogenase (SDH) or fumarate hydratase (FH) is reduced may result in tumorigenesis [9,10]. Indeed, the *SDH *and *FH *genes have been demonstrated to be tumour suppressor genes (TSG) via this pseudohypoxic drive in paraganglioma [11], hereditary leiomyomatosis and renal cell carcinomas [7]. In view of this potential mechanism to enhance HIF-1α levels and in view of the association of HIF-1α levels with breast cancer prognosis and resistance to treatment, we hypothesised that epigenetic silencing by promoter methylation for the *SDH *and *FH *genes may be a mechanism underlying upregulated HIF-1 in a proportion of breast carcinomas.

## Materials and methods

### DNA samples

DNA was obtained from 72 invasive breast carcinomas from the John Radcliffe Hospital, Oxford, UK (Ethics committee approval: JR C02.216) and from the following cancer cell lines: breast: MCF10A, MCF7, BT20, SkBr3, Hs578T, T47D, MDA-MB 153, MDA-MB 468, MDA-MB 453, MDA-MB 231; colorectal: Colo205, HCT116, SW948, SW48; leukaemia: HL60, KG1, RPMI8226, CCRF-CEM; ovarian: 2008; neuroblastoma: SK-N-SH, SH-SY5Y, Be(2)c, IMR32; and prostate: PC3.

### Bisulfite modification

DNA from samples were bisulfite modified as described previously [12]. CpGenome™ Universal Methylated DNA (Chemicon/Millipore, Billerica, MA) and DNA from peripheral blood mononuclear cells were used as the methylated and unmethylated controls, respectively. Standards (5, 10, 25 and 50% methylation) were generated by diluting Universal Methylated DNA in the unmethylated DNA. Whole-genome amplification (WGA) DNA was used as an alternative unmethylated control [12].

### Methylation-sensitive high resolution melting (MS-HRM) and methylation-specific PCR (MSP)

Methylation-sensitive high resolution melting (MS-HRM) was performed on bisulfite modified DNA [13]. MS-HRM primer sequences and optimal annealing temperatures are listed in Table 1. Bisulfite modified fully methylated, peripheral blood DNA, WGA DNA, different methylation percentage standards and no template controls were used in each run as controls and standards. Assays were performed in duplicate.

**Table 1:** Primer sequences, annealing temperature and amplicon information for the MS-HRM assays.

**Gene**	**Primer Sequences** **5' - 3'**	**Annealing temperature (°C)**	**Amplified region (GenBank accession and nucleotide numbers)**	**Screened CpGs/amplicon size (bp)**
*SDHA*	F - CGGGGTTTTAAAAATGTTGGTGTT	61	AC021087.5: 218153-218484	39/332
	R - CGAACCCCCGACATATCTACTATTACC			
				
*SDHB *1	F - CGGGGGAAGTTAAATGGGTATG	60	AL049569.13: 17380588-17380744	14/157
	R - CGCCCAACCTACATCCACTAAA			
				
*SDHB *2	F - GCGGTTAGTGGGTTTTTAGTGGAT	65	AL049569.13: 17380446-17380623	16/178
	R - CAAACAAACTCCGCCAAAAATTATAACC			
				
*SDHC*	F - TCGTTATATGATATTTTTAATTTCGATTTTTAGT	56	AL592295.25: 161284096-161284197	8/102
	R - ATCTTAAATTCCGATCTAAACGAAAATAAC			
				
*SDHD*	F - CGGGTTGGTGGATGATTTTGAG	62	AP002007.4: 111957596-111957689	4/94
	R - CCTCACCTCGACCTCCTAAAACAC			
				
*FH*	F - TTTGTTTTATTTGTCGGTGTGAGGT	60	AL591898.1: 241683032-241683154	7/123
	R - AAAACTTAAATAAAATTTCTAAACGACTATAACCAC			

**Figure 1:**
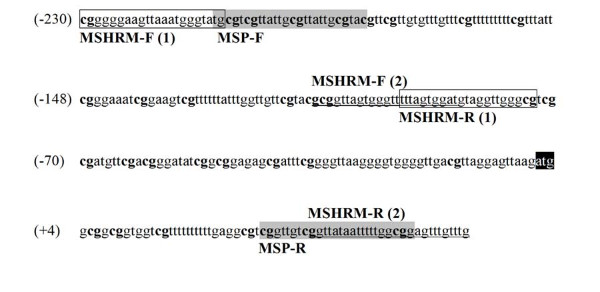
***SDHB *bisulfite modified promoter sequence with primers indicated**. *SDHB *MS-HRM (1) primer sequences are shown in boxes and *SDHB *MS-HRM (2) primers are underlined. *SDHB *MSP primer sequences are shaded in grey. CpG dinucleotides are in bold. The number in parentheses on the left is the nucleotide position relative to the starting codon ATG (shaded in black).

Methylation-specific PCR primer sequences and PCR conditions of *SDHB *methylation were described previously in [14]. The positions of the *SDHB *MS-HRM and MSP primer sequences in the *SDHB *promoter sequence are shown in Figure 1.

## Results

### Methylation of SDHA, SDHB, SDHC, SDHD and FH in cell lines and tumours

**Figure 2:**
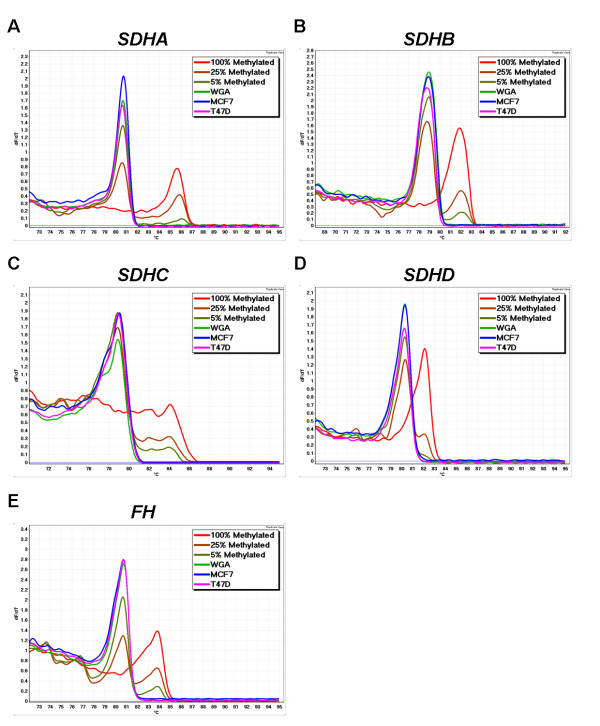
***SDH *and *FH *MS-HRM in breast cancer cell lines**. In MS-HRM, PCR products undergo melting analysis after PCR amplification. Different amplicons have different melting behaviours according to their methylation status. Unmethylated samples melt earlier than methylated samples as they have unmethylated cytosines replaced by thymines in the sequence. Controls for 100%, 25%, 5% methylation and WGA are shown. Levels of methylation as low as 5% can be readily seen. The cell lines shown here, MCF7 and T47D showed no methylation of the four *SDH *subunits and *FH*. A. *SDHA*; B. *SDHB*; C. *SDHC*; D. *SDHD*; and E. *FH *methylation. The curve for each sample represents the mean value of duplicate samples.

**Figure 3:**
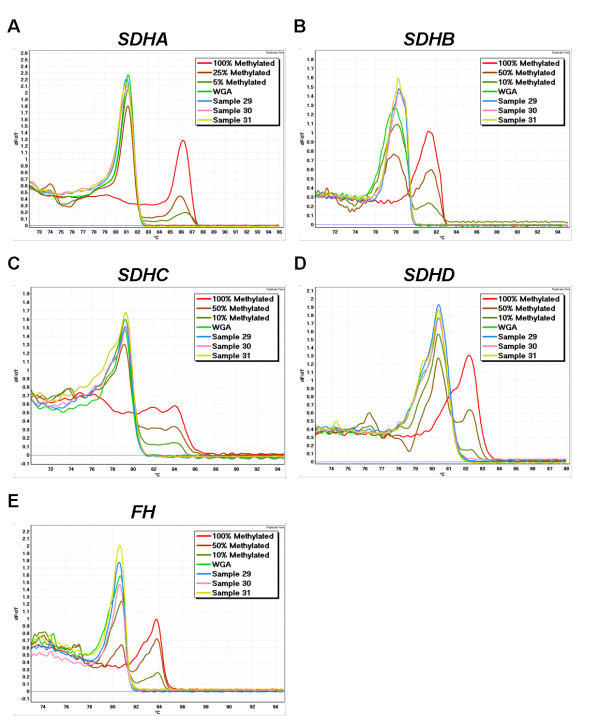
***SDH *and *FH *MS-HRM in breast carcinoma samples**. All the breast carcinoma samples showed no methylation. Three representative cancers are shown. The curve for each sample represents the mean value of duplicate samples.

CpG islands were identified in the promoter region of *SDHA, SDHB, SDHC, SDHD *and *FH *demonstrating the potential for alteration of their gene expression by methylation. MS-HRM primers were designed to cover CpG rich areas of the proximal promoter region for each gene. However, methylation was not observed for any of the 5 genes in any of the 10 cell lines tested (Figure 2) or in any of the 72 invasive breast carcinomas (Figure 3).

**Figure 4:**
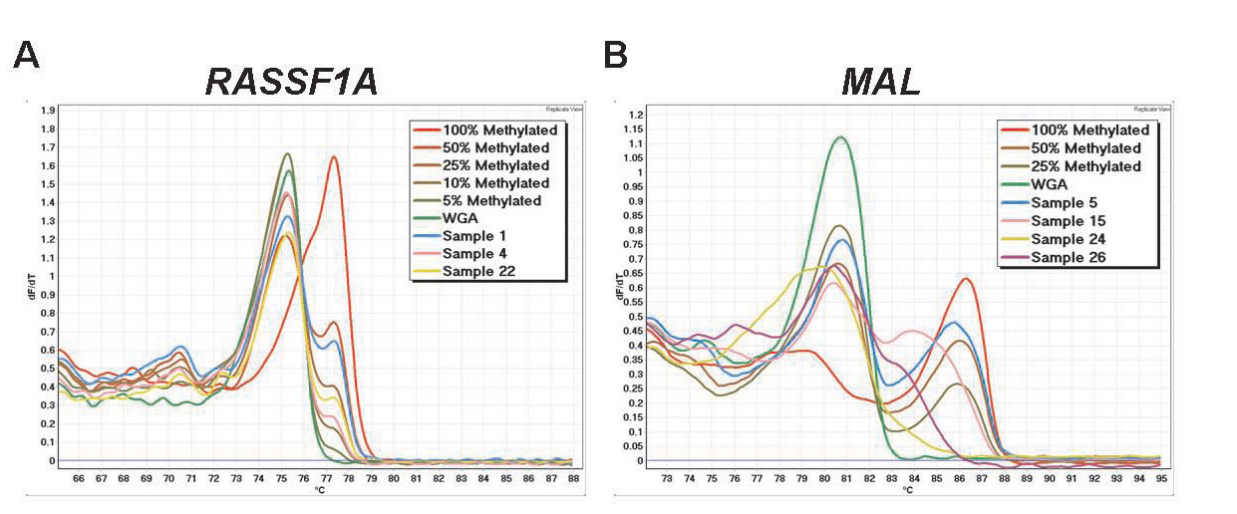
***RASSF1A *and *MAL *MS-HRM of breast carcinoma samples**. A. *RASSF1A *MS-HRM demonstrates generally homogeneous methylation in the breast carcinoma samples at different percentages of methylation ranging from 10 to 50% methylation. B. Both homogeneous (the sharp peak seen for sample 5) and heterogeneous methylation (the more complex melting profiles across both unmethylated and methylated peaks seen in the other samples) can be observed for *MAL*.

The absence of detectable methylation was not due to technical reasons such as absence of breast cancer material. MS-HRM assays for two genes known to be methylated in a large proportion of breast cancers were used as controls to show that methylated genes could be detected in the 72 breast carcinoma samples using our methodology. A high frequency of *RASSF1A *(83.3%) and *MAL *(41.7%) methylation was identified in the breast carcinomas [15,16]. Only ten breast carcinoma samples showed no methylation for either control markers (Table 2). *RASSF1A *showed principally homogeneous methylation whereas *MAL *showed mostly heterogeneous methylation in these 72 invasive breast carcinomas (Figure 4). These results indicate methylation of the *SDHA, SDHB, SDHC, SDHD *and *FH *genes in these samples would have been detected if it was present.

**Table 2:** *RASSF1A *and *MAL *methylation frequencies in the breast carcinoma samples as determined by MS-HRM

	***RASSF1A *Positive**	***RASSF1A *Negative**	**Total**
***MAL *Positive**	28 (38.9%)	2 (2.8%)	30 (41.7%)
***MAL *Negative**	32 (44.4%)	10 (13.9%)	42 (58.3%)
**Total**	60 (83.3%)	12 (16.7%)	72

## Discussion

Overexpression of HIF-1α has been previously reported to correlate with angiogenesis [5], an aggressive phenotype [3,17] and poor outcome after conventional adjuvant therapy [18,19] in breast cancer. Thus mechanisms that enhance HIF-1α expression are important in cancer development and would be potential targets for treatment [2,20].

The tricarboxylic acid cycle enzymes, SDH and FH are involved in the conversion of succinate to fumarate and fumarate to malate, respectively. SDH also takes part in the electron transport chain as a functional complex II member.

Both SDH and FH can act as tumour suppressors, and germline mutations in their genes predispose to tumour development. Mutations in the genes coding for SDH subunits *B*, *C *and *D *predispose to familial paragangliomas and phaeochromocytomas [11,21,22], and mutations in *FH *cause hereditary leiomyomatosis and renal cell carcinomas [23].

Although the mechanisms that link *SDH *and *FH *mutations to tumour formation are unclear, it is likely that pseudohypoxia is a primary mechanism. Both Selak *et al*. [8] and Pollard *et al*. [24] have suggested that overexpression of HIF-1α in normoxic conditions is due to the accumulation of succinate, which then is able to inhibit the activity of HIF-1α prolyl hydroxylases via product inhibition. A recent study has also shown that disruption of mitochondrial metabolism using small interfering RNAs to silence *SDHB *resulted in up-regulation of HIF-1α. [25]. Furthermore, microarray analysis has confirmed that genes involved in the hypoxic pathway are dysregulated when *SDHB *is silenced [25].

Since many tumour suppressor genes are known to be inactivated by DNA promoter methylation, we examined promoter methylation of *SDH *and *FH *in a cohort of breast carcinomas. However, we found no evidence of DNA methylation of the promoter regions of these genes in breast carcinomas cancer or a panel of cancer cell lines, including ten breast cancer cell lines, making it unlikely that methylation of the promoter regions of these genes is responsible for increased HIF expression in breast cancers. Although we cannot exclude the possibility that methylation of regions other than the proximal promoter may be involved, our findings are also in keeping with others who have been unable to demonstrate methylation of *SDHD *in neuroblastomas and *FH *in renal cell cancers [26,27].

**Figure 5:**
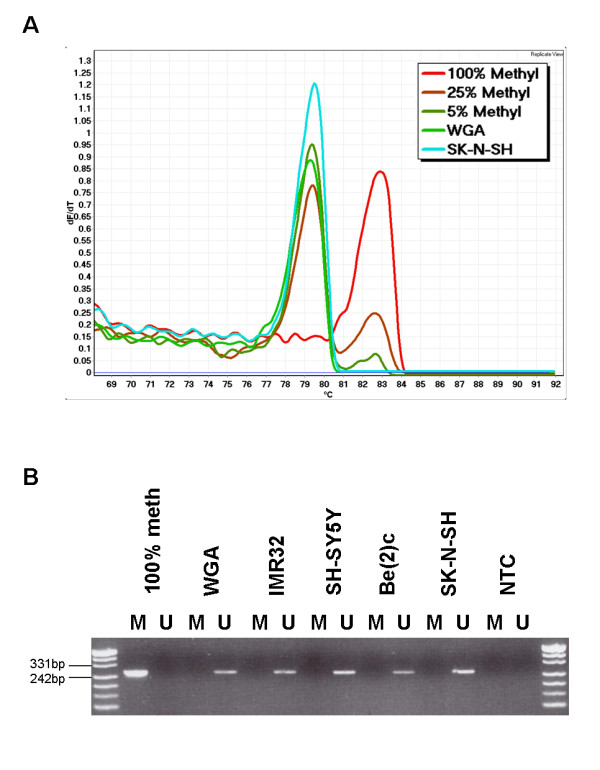
***SDHB *MS-HRM and MSP analysis in neuroblastoma cell lines**. A. *SDHB *MS-HRM of the SK-N-SH neuroblastoma cell line; the early melting shows that *SDHB *is clearly unmethylated in the SK-N-SH cell line isolate used by us (the curves for each sample represents the mean value of duplicate samples). B. Four bisulfite modified neuroblastoma DNA (IMR32, SH-SY5Y, Be(2)c and SK-N-SH) were amplified using specific *SDHB *methylated (M) and unmethylated (U) MSP primer pairs. Fully methylated DNA (100% methyl) and fully unmethylated DNA (WGA) were used as controls. The no template control (NTC) was also included as a negative control for both methylated and unmethylated PCR.

Promoter methylation of *SDHB *has been previously reported in primary sporadic phaeochromocytoma (32%) and neuroblastoma (21%) [14]. We were unable to demonstrate the previously reported *SDHB *promoter methylation in the SK-N-SH neuroblastoma cell line [14] using both MS-HRM and methylation-specific PCR (MSP) assays (Figure 5). Since MS-HRM methodology is capable of detecting levels of methylation as low as 5%, it suggests that the methylation-specific PCR (MSP) that was previously used may have miscalled the methylation. MSP is prone to false positives, particularly if incomplete conversion is present. The reported absence of correlation between the apparent *SDHB *methylation and *SDHB *gene expression in the cell line used in that study further supports the possibility that the methylation was artefactual [14].

In conclusion, promoter methylation of the *SDHA, SDHB, SDHC, SDHD *and *FH *genes is unlikely to be an important mechanism in stabilising HIF-1 in breast carcinomas through the downregulation of the expression of *SDH *and *FH *genes.

## Competing interests

The authors declare that they have no competing interests.

## Authors' contributions

KTH participated in the design of the experiment, performed the experiments and data analysis and drafted the manuscript. AD participated in the design of the experiment and helped to analyse the data. SBF conceptualised the study and supervised the work. All authors contributed to the manuscript and read and approved the final draft.
